# Reproduction in Trypanosomatids: Past and Present

**DOI:** 10.3390/biology10060471

**Published:** 2021-05-27

**Authors:** Camino Gutiérrez-Corbo, Bárbara Domínguez-Asenjo, María Martínez-Valladares, Yolanda Pérez-Pertejo, Carlos García-Estrada, Rafael Balaña-Fouce, Rosa M. Reguera

**Affiliations:** 1 Departamento de Ciencias Biomédicas, Facultad de Veterinaria, Universidad de León, 24071 León, Spain; mgutc@unileon.es (C.G.-C.); bdoma@unileon.es (B.D.-A.); myperp@unileon.es (Y.P.-P.); cgare@unileon.es (C.G.-E.); rmregt@unileon.es (R.M.R.); 2 Instituto de Ganadería de Montaña CSIC-Universidad de León, 24346 León, Spain; mmarva@unileon.es

**Keywords:** Trypanosomatids, clonal and sexual reproduction, genetic exchange, ploidy, virulence, drug resistance

## Abstract

**Simple Summary:**

The reproduction of trypanosomatids is a fundamental issue for host–parasite interaction, and its biological importance lies in knowing how these species acquire new defense mechanisms against the countermeasures imposed by the host, which is consistent with the theory of the endless race or the Red Queen hypothesis for the existence of meiotic sex. Moreover, the way these species re-produce may also be at the origin of novel and more virulent clades and is relevant from a thera-peutic or vaccination point of view, as sex may contribute to increased tolerance and even to the rapid acquisition of drug resistance mechanisms. Kinetoplastids are single-celled organisms, many of them being responsible for important parasitic diseases, globally termed neglected diseases, which are endemic in low-income countries. Leishmaniasis, African (sleeping sickness) and American trypanosomiasis (Chagas disease) caused by trypanosomatids are among the most ne-glected tropical scourges related to poverty and poor health systems. The reproduction of these microorganisms has long been considered to be clonal due to population genetic observations. However, there is increasing evidence of true sex and genetic exchange events under laboratory conditions. We would like to highlight the importance of this topic in the field of host/parasite in-terplay, virulence, and drug resistance.

**Abstract:**

Diseases caused by trypanosomatids (Sleeping sickness, Chagas disease, and leishmaniasis) are a serious public health concern in low-income endemic countries. These diseases are produced by single-celled parasites with a diploid genome (although aneuploidy is frequent) organized in pairs of non-condensable chromosomes. To explain the way they reproduce through the analysis of natural populations, the theory of strict clonal propagation of these microorganisms was taken as a rule at the beginning of the studies, since it partially justified their genomic stability. However, numerous experimental works provide evidence of sexual reproduction, thus explaining certain naturally occurring events that link the number of meiosis per mitosis and the frequency of mating. Recent techniques have demonstrated genetic exchange between individuals of the same species under laboratory conditions, as well as the expression of meiosis specific genes. The current debate focuses on the frequency of genomic recombination events and its impact on the natural parasite population structure. This paper reviews the results and techniques used to demonstrate the existence of sex in trypanosomatids, the inheritance of kinetoplast DNA (maxi- and minicircles), the impact of genetic exchange in these parasites, and how it can contribute to the phenotypic diversity of natural populations.

## 1. Clonal Theory or True Sex?

The question of how trypanosomatids reproduce is the subject of debate between two often apparently irreconcilable schools of thought. On the one hand, there are those who, based on the findings of population genetics, have defended for many years the asexual reproduction or the predominant clonal evolution of these unicellular eukaryotic parasites. On the other hand, there are those who, aided by molecular tools, defend the sexuality of these species as an alternative mode of reproduction based on the results of experimental studies of hybridization in the laboratory. It is now known that some microbial parasites whose reproduction has been classically explained with the clonal theory, are capable of sexual reproduction, including *Plasmodium*, a parasite with an obligatory sexual cycle [1].

For years, the clonal theory was the only model that explained the reproduction of trypanosomatids and many other species of parasitic protozoa, as it provided evidence of genomic stability of these microorganisms over generations. In addition, gene polymorphism studies indicated that segregation and recombination processes, which are typical indicators of sexual reproduction, appeared at low frequency or were absent in natural populations of these species [2]. According to this theory, mitotic division by itself is capable of forming filial clones identical to the parental during generations, in which the only evolutionary force is the introduction of random mutations and the consequent natural selection of optimal genotypes. These changes could increase the interactive fitness of the parasite with the host or, conversely, could be deleterious, thus favoring the prevalence of mutated strains that could lead to their extinction according to Muller’s ratchet principle [3,4]. In the early 1990s, Tibayrenc and co-workers supported the strict clonal reproduction model based on the apparent absence of two fundamental features of sexual reproduction in natural populations of trypanosomatids, namely segregation and recombination [2,5]. Gene segregation implies that during gamete formation each locus of the genome is separated from its homologue, this process giving rise to one allele for each gene. Recombination refers to the relationship between the alleles at different loci after mating [2]. One evidence that supported the absence of segregation in these microorganisms was the presence of constant heterozygosity (unlike sexual reproduction, which generates both homozygous and heterozygous individuals) in the same loci, and the tendency of clonality to modify the expected distribution of genotypes, thus producing deviation from Hardy–Weinberg equilibrium [6,7,8,9].

Furthermore, a number of observations based on population genetics supported the strict clonal theory: (i) all populations studied showed a strong imbalance in allele frequencies, regardless the stage of life cycle considered; (ii) all populations showed almost total absence of recombinant genotypes; (iii) an over-representation of dominant genotypes distributed over wide geographical areas was observed, and (iv) a clear association between unrelated genetic markers was also detected [10]. The deviation from Hardy–Weinberg equilibrium of field populations could illustrate the low Mendelian inheritance and the stable increase in the occurrence of heterozygosity over generations due to the absence of recombination. Therefore, in the absence of strong evidence of genetic exchange, and despite the fact that other protists had sexual reproductive stages with haploid gametes present (as is the case of the Phylum Apicomplexa), strict clonality was a very tempting option to fully explain trypanosomatids reproduction.

In the early 1970s, evolutionary researchers launched an appealing theory that tried to explain the co-evolution of parasite/host species, proposing that two species evolving together were engaged in an endless race to acquire weapons and countermeasures in order not to be eliminated by the other species. This hypothesis (arms race dynamics or “Red Queen” theory) put sex as one of the mechanisms involved in the rapid acquisition of effective tools by both the parasite and the host in response to changes introduced by the species co-evolving with [11,12]. Sex, despite its disadvantages such as slower reproduction rate, waste of energy in gamete formation, and the need for a male, has a greater adaptive capacity to allow both species to continue in the race. Moreover, sexual reproduction has been linked to the evolution of eukaryotes since their common ancestor [13,14,15]. In other species of unicellular eukaryotes this debate is also ongoing, and it is important to evaluate in parallel the findings of the different species, such as the similarities found between fungi and pathogenic parasites. The relationship and parallelism between mainly clonal population structures and modes of reproduction including sexuality among these types of eukaryotic microbes, have also been of interest to some authors [16,17,18]. A recent review compiles the most significant advances in the three major groups of parasitic protists (Apicomplexa, Excavata, and Amoebozoa) [19].

The lack of adequate methodologies to evaluate the results of experimental crosses under laboratory conditions, and certain features of trypanosomatids biology did not facilitate the proposal of valid alternative hypotheses to the theory of predominant clonal evolution. Some of these features are: (i) these species are mainly diploid species, but this was not demonstrated experimentally until the introduction of adequate molecular biology techniques. In fact, the percentage of strictly diploid cells is very low within a population, as each of their chromosomes can be present in more than one ploidy state [20,21,22] as it occurs in some fungal species [23]; (ii) another unique feature of these parasites is their inability to condense chromosomes during any phase of the cell cycle, thus impeding their visualization during mitosis and/or meiosis; (iii) light and/or electron microscopy are not able to distinguish haploid gametes from normal cells, or the fusion of their cytoplasm (syngamy/plasmogamy) or nuclei (karyogamy) during mating; (iv) finally, the genetic toolkit required for meiosis has been discovered recently in these microorganisms [24,25,26].

The first laboratory experiments that challenged the strict clonal theory as the sole model of reproduction in trypanosomatids were conducted by Jenni and Tait in the early 1980s using *T. brucei* isoenzyme markers [27]. These early phenotype-based studies were later complemented by the comparison of genome restriction maps of parental and hybrid populations, the introduction of drug resistance markers, and the use of fluorescent transgenic strains.

Since then, a lot of evidence of genetic exchange in trypanosomatids has confirmed that recombination processes are much more frequent than previously thought, and have led to rewrite the theory of “strictly clonal reproduction” [28,29] and replace it with that of “predominant clonal evolution”, in which genetic recombination processes fit, but are strongly restricted in order not to break the clonal population pattern. The main points of the predominant clonal theory are: (i) the existence of a strong linkage disequilibrium in the population; (ii) the occurrence of episodes of genetic exchange leading to the emergence of stable genetic clusters called “near-clades” [30], and (iii) the existence of a “clonal threshold” above which prevailing clonal evolution would counteract genetic recombination, or a definitive genetic clade would be established [28,31]. However, there are disagreements with other authors regarding some of these points. For example, some researchers indicate that linkage disequilibrium is not an accurate indicator for determining the rates of sexual and asexual reproduction in a population [32]. Moreover, inadequate sampling may increase the likelihood of drawing erroneous conclusions about the Wahlund effect [6,22,33]. Other authors consider that it is difficult to fully explain the high genetic diversity of kinetoplastids by the predominant clonal evolution model [34].

**Figure 1:**
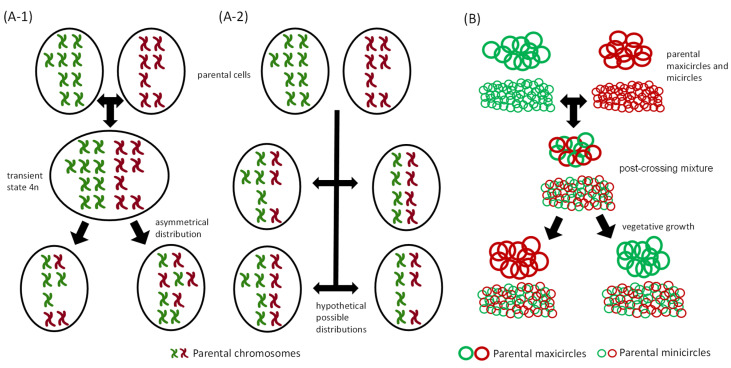
Genomic and kDNA inheritance in trypanosomatids: (**A**) Models representing two possible sexual reproduction strategies in trypanosomatids that allow mixture of parental genotypes in daughter cells: (**A-1**) A parasexual process in which two aneuploid parental cells (approximately 2n) are fused to give rise to a transient aneuploidy genotype (approximately 4n), which further reduces its chromosomal burden in an asymmetric way to produce aneuploid daughter cells (approximately 2n). (**A-2**) Meiotic process, in which the hypothetical putative distribution of parental chromosomes is represented in daughter cells. Cells are represented with 4 pairs of chromosomes and reflect the mosaic aneuploidy that is frequent in the trypanosomatid *Leishmania.* A classical image of chromosomes is depicted in the figure, despite the fact that the genetic material does not condense during the life cycle of trypanosomatids. Chromosomal recombination between homologous chromosomes is not represented in the figure; (**B**) model representing the result of kDNA inheritance. It can be biparental in maxicircles and minicircles, but after some clonal divisions, maxicircles, unlike minicircles, only maintain one parental genotype.

It is widely accepted that there is genetic exchange in trypanosomatids, but the discussion is now centered on its impact on population structure. The controversy focuses on whether the impact of genetic recombination is on an evolutionary scale, as stated by Tibayrenc and Ayala, or if, on the contrary, the impact produced is much greater, more frequent, and with a high epidemiological effect [21,22,34,35]. The potential of next-generation sequencing (NGS) systems, as well as the use of bioinformatics tools such as PAINT [36], may help solve these and other questions about the impact of genetic exchange and reproductive strategies in these parasites (Figure 1A).

## 2. Genetic Exchange in *Trypanosoma*

Prior to the publication of the major trypanosomatids genomes and the use of rapid DNA sequencing tools, experimental evidence supporting sexual reproduction in these species was scarce. Microscopic studies were inconclusive in elucidating the existence of meiosis, gamete formation and karyogamy. It was not until there was sufficient experimental evidence from reliable phenotypic studies that the existence of genetic exchange events in trypanosomatids could be used as alternative to clonality [27,37]. Tait in 1980, using selected housekeeping isozymes, not only demonstrated the diploid nature of *T. brucei*, but also showed that the proportion of allelic frequencies of isozyme markers corresponded with a high degree of heterozygosity, which led to the conclusion that these populations were in Hardy–Weinberg equilibrium [27,37]. These results were later confirmed by Gibson and co-workers in 1985, who not only provided new clues of the diploid nature of *T. brucei* using restriction fragment length polymorphisms (RFLP) around three glycolytic enzyme coding genes and the tubulin gene cluster, but also proved the existence of gene exchange in different natural populations of *T. brucei*. However, the authors were unable to conclude whether the combinations of the genetic markers studied were the result of chromosomal rearrangement, or whether they corresponded to a recombination between homologous chromosomes [38].

The first generation of experimental hybrids from two genetically distinct parental strains of *Trypanosoma* (*T. brucei brucei* and *T. brucei gambiense* type II) was obtained by Jenni and co-workers in 1986. The hybrid progeny, isolated from tsetse flies, was analyzed both phenotypically and genotypically and showed that the first filial generation was heterozygous for the parental markers used. In addition, it suggested the possibility, for the first time, that the parental strains underwent a meiotic process followed by the fusion of haploid gametes within the tsetse fly. Finally, the authors suggested that hybrid genotypes were produced not only under laboratory conditions, but were also present in field isolates, thus indicating that this was a naturally-occurring process [39]. In 1987, Wells and co-workers using the same isolates as diploid parental strains, showed that the DNA content of the hybrid progeny was much higher than that of the parental, and was stable over time under laboratory conditions and after mouse passage. These experiments confirmed for the first time the formation of polyploids as a consequence of hybridization [39].

One important step forward in the study of gene exchange in trypanosomatids was the use of reverse genetics techniques. For this purpose, Gibson and Wittington (1993) created two parental strains of *T. b. brucei* and *T. b. rhodesiense* by stably transfecting two heterologous genes conferring resistance to geneticin and neomycin. Then, *Glossina* flies fed with infected bloodmeal were dissected and salivary glands and midgut were used to isolate procyclic trypanosomes. Although the number of hybrids displaying dual drug resistance was low, they were found exclusively in salivary glands and had different genotypes, many of them being trisomic [40,41,42,43]. Additional evidence that the genetic exchange in *T. brucei* follows Mendelian inheritance and is mediated by meiosis was obtained by analyzing the inheritance of 11 microsatellite and minisatellite markers located on different chromosomes of *T. b. brucei* and *T. b. gambiense* after mating. Genetic analysis showed that the alleles of the progeny had been segregated according to the expected mendelian frequencies, and that the alleles located on different chromosomes were assorted independently, which provided unequivocal proofs that genetic exchange events had taken place [44].

A further advance in the study of the sexual reproduction of trypanosomatids was the introduction of fluorescent biomarkers. A *T. brucei* strain expressing GFP under the control of a tetracycline (Tet)-inducible expression system [45] was used to demonstrate meiotic segregation and gene rearrangement after mating [46]. The experimental cross was carried out using two strains of *T. brucei*; a clone stably transfected with the GFP-encoding gene at the *ssu* (18S-rRNA) locus and (Tet) stably integrated in trans on a different chromosome (strain K11), and a wild-type clone. Since GFP-emitting trypanosomes resulted from the release of the repressor (Tet) due to hybridization, it was concluded that the resulting segregation and rearrangement of chromosomes carrying the GFP and Tet gene markers were consistent with the prediction of Mendelian inheritance and meiosis [43].

A simple and efficient alternative consisted of observing the resulting phenotype of a crossing between two transgenic parental cell lines of *T. brucei*, one carrying the GFP-coding gene and the other the red fluorescent protein (RFP)-coding gene, both integrated at the *ssu* locus. Yellow clones resulting from the emission of both fluorescent proteins in the hybrid phenotype were visualized after mating in salivary glands. Genetic analyses showed that the hybrid progeny had a biparental inheritance from the nuclear genomes, but the recovery of a large proportion of polyploid, probably the product of unreduced gametes during meiosis, challenged the mendelian inheritance as the only way to explain the non-clonal reproduction of these microorganisms [47].

This experimental design was used one year later to demonstrate that intraclonal crossing (self-fertilization or selfing) was feasible using a unique *T. b. brucei* parental cell line, which had been previously modified to express GFP and RFP. After crossing, it was found that frequency of recovery of yellow hybrids was roughly half of what is expected when the crossing is done between two different *Trypanosoma* strains. To explain this phenomenon the authors postulated that a proportion of trypanosomes produced non-viable diploid hybrids after mating. An interesting possibility would be that trypanosomes could somehow recognize self from non-self. This would favor the viability of diploid hybrids coming from crosses between different strains rather than intraclonal ones that would die, thereby pointing to the existence of “mating types” in trypanosomes (see below) [48].

Once gene segregation and recombination had been demonstrated, it remained to be determined whether or not trypanosomes produced gametes, how meiosis was produced, and the toolkit to trigger it. Soon after the release of the genomes of the three major kinetoplastids (*T. cruzi*, *T. brucei* and *Leishmania*), Malik and coworkers in 2007 [49] described in the *Trichomonas vaginalis* genome the existence of eight genes—*hop1*, *hop2*, *mnd1*, *dmc1*, *msh4*, *msh5,* and *mer3*—involved in the meiosis of diploid organisms. These genes are also present in the *T. brucei* genome [49]. Although *T. vaginalis* and kinetoplastids were previously included within Excavata supergroup, they are now classified within different categories; *Trichomonas* within Discoba and kinetoplastids within Metamonada [50]. The functionality during the prophase of meiosis of the four homologous recombination genes *spo11*, *mnd1*, *dmc1* (involved in processing DNA during recombination), and *hop1* (a part of synaptonemal complexes) was analyzed by generating transgenic *T. brucei* cell lines containing these genes fused to the yellow fluorescent protein (YFP). Gene expression, visualized by the yellow fluorescence emitted by the meiotic protein, occurred in the salivary glands of flies, and both the order of expression of these genes and the role of their proteins were similar to those in other eukaryotic species [51]. It has been reported that heat shock leads to an increase in the abundance of several mRNAs, including some specific meiotic genes in *T. brucei* [52], although the induction of a meiotic cycle triggered by stress factors has not yet been demonstrated in trypanosomatids.

The existence of haploid gametes isolated from *Glossina* salivary glands was shown using transgenic strains of *T. brucei* expressing GFP and RFP. Microscopical observation of the interaction and fusion of cytoplasms (syngamy), could be visualized as a mixture of green and red colors from each parental clone, thus giving rise to yellow/orange (merge) hybrids. It is noteworthy that flagella of the gametes intertwined during mating as an approximation maneuver to facilitate surface interaction between the cells. The total nuclear DNA content of these cells—measured as total fluorescence intensity of the nucleus—revealed that these promastigotes emitted half as much fluorescence as their parental line, which indicates that they were haploid compared to diploid metacyclic parasites [53]. The formation of the gametes began shortly after the contact of red and green fluorescent cells, both being of similar and different genotypes. However, after numerous fusion experiments, the authors hardly found differences in the mating of haploid gametes and their interactions when trypanosomes were mixed intra- or interclonally. Nevertheless, the exchange of their cytoplasms occurred to a lesser extent in intraclonal crossing experiments than in interclonal ones [54]; however, despite the differences found in mating between intra and interclonal crosses, nothing is yet known about the molecular basis underlying this process. A model of meiosis in *T. brucei* has recently been proposed. In this model, the meiotic intermediaries and gametes that participate sequentially in the sexual cycle are described. The order of events was determined by the differences existing in nuclear and kDNA content between cell types and by the expression of the *hap2* gene, which occurs in gametes prior to cell fusion, and in the case of *T. brucei*, in some meiotic intermediaries as well [55].

In other Trypanosomes, like *Trypanosoma cruzi*, the etiological agent of Chagas disease, some studies have described populations of this parasite as being predominantly clonal [7,28]. Nevertheless, other studies have suggested that recombination phenomena and sexual reproduction may be quite frequent [56,57,58,59,60]. In addition, it has also been suggested that in some niches highly clonal parasite populations (with a past of genetic exchange) may coexist with other populations that exhibit frequent sexual reproduction [60]. It is now widely accepted that some Discrete Typing Units (DTUs) of *T. cruzi* are hybrids originating from genetic exchange events occurred in the past, although it is still under discussion whether one or more hybridization events took place in the evolutionary history of *T. cruzi* [59,61,62,63]. A single successful experiment of an experimental cross [64], in which two parents of the TcI clade that are resistant to two different drugs were co-cultured in mammalian host cells, gave rise to hybrid progeny with six double-resistant isolates. These hybrids were analyzed and showed a subtetraploid genotype, which contained 15.3% less DNA than a true tetraploid. These data seem to fit with a process in which the fusion of two diploid parents would form a tetraploid that would subsequently undergo allelic loss, thereby resulting in stable hybrid progeny between triploidy and tetraploidy [64,65].

## 3. Genetic Exchange in *Leishmania*

First evidence provided by restriction site polymorphisms, gene silencing, gene recombination, and karyotype alterations, suggested that the predominant state of *Leishmania* was the diploidy [66]. However, since then, there is growing evidence that natural populations of *Leishmania* are often aneuploid, and this parasite has a special ability to tolerate changes in chromosome number [67]. This aspect was described during an attempt to create null mutants of *L. major* for the dihydrofolate reductase-thymidylate synthase (*dhfr-ts*) locus by Cruz and coworkers [68], who obtained mainly aneuploid and tetraploid clones for this gene. Similar results were obtained with other genes encoding housekeeping enzymes [69]. To explain the low frequency of heterozygotes (absence of segregation) observed in natural populations of *Leishmania*, a state of “transitory aneuploidy” that could generate homozygous and heterozygous progeny from heterozygous parents was proposed. Gueiros-Filho and Beverley (1996) indicated that the rate of spontaneous loss of heterozygosis for the *dhfr-ts* locus was 10^−5^ cells/generation during selection against heterozygous lines, which points to “transient aneuploidy” as a mechanism to maintain homozygosis in *Leishmania* populations [70]. When Lachaud and co-workers analyzed in 2014 the ploidy of three Old World *Leishmania* spp. (*L. infantum*, *L. donovani,* and *L. tropica*), and one from the New World (*L. amazonensis*) by fluorescent *in situ* hybridization (FISH), they found an aneuploid mosaic for six different chromosomes, which pointed out this feature as a general characteristic of the genus *Leishmania* [71]. This does not seem to be just restricted to this trypanosomatid, since the existence of widespread aneuploidy has also been reported in the *T. cruzi* genome [71]. This generalized aneuploidy would contribute to maintain the genotypic diversity without the need of gametes, whose existence in *Leishmania* has not yet been demonstrated.

The existence of natural hybrid genotypes in *Leishmania* had been previously described in field and clinical isolates. In 1991, Kelly and co-workers characterized two hybrid strains of *Leishmania* isolated from wild animals in Saudi Arabia through isoenzyme and karyotype studies, which showed mixed characteristics of both *L. major* and *L. arabica* species [72]. Other field studies with natural isolates from the New World confirmed the existence of hybrids between *L. braziliensis* and *L. panamensis* in Nicaragua [73], and *L. braziliensis* and *L. peruviana* in Peru [29,74]. In addition, the first indication for the existence of hybrids between two more genetically distant species and with different tissue tropism was described by Ravel and co-workers in 2006, using *L. infantum* and *L. major* obtained from clinical isolates of immunocompromised patients in Portugal [75]. Later on, the ability of these hybrids to infect permissive and selective sand fly vector species *Lutzomya longipalpis* and *Phlebotomus papatasi* (selective for the transmission of *L. major* but not *L. infantum*), respectively, was also studied [76]. Since hybrids could be recovered from both permissive and non-permissive vectors, it was concluded that characters inherited from *L. major* were responsible for the colonization of the selective sand fly vector and could underlie its expansion into new geographic niches [76].

Mass sequencing technology was used to quantify the frequency of sexual reproduction in eleven *L. infantum* isolates obtained from an outbreak in Cukurova region (Turkey), where data from visceral leishmaniasis (VL) cases have not been currently reported. The population sampled descended from a hybrid strain of *Leishmania*, produced from a single cross between a parent related to *L. infantum*, and a second parent, most likely related to *L. donovani*. The irregular pattern of heterozygosity obtained suggested that these strains resulted from a cross between two genetically different species of the *L. donovani* complex. After hybridization, the population would have reproduced by binary fission, although occasional recombination events would have taken place, albeit with a low frequency. These results show that behind the epidemic episodes of leishmaniasis in a specific geographical focus, there can be genetic exchange phenomena between populations or even between different *Leishmania* species [77].

The first empirical demonstration that *Leishmania* was able to undergo sexual reproduction resulting from interspecific and intraspecific crossings was presented by Akopyants and co-workers in 2009. These authors crossed two parental lines of *L. major*: LV39c5 (HYG), heterozygous for an allelic replacement of the LPG5A on chromosome 24 by a hygromycin B resistance cassette, and the modified strain of *L. major* Friedlin V1, FV1 (SAT), the latter containing a heterozygous nourseothricin resistance (SAT) marker integrated along with a linked firefly luciferase (LUC) reporter gene into one allele of the 24S ribosomal RNA locus of chromosome 27. The recovered hybrid progeny had inherited alleles from both parents, thereby showing a heterozygous genotype [78]. Inbar and co-workers, using parental clones of *L. major* bearing independent drug markers, analyzed the crossing success and the time required to obtain hybrids both in *Ph. duboscqi*, the natural vector of *L. major*, and in the non-natural but permissive *Lu. longipalpis* vector. Hybrids were recovered from the mid-gut of both fly species with similar efficiencies, which suggests that *L. major* lacks “mating types” that limit free genetic exchange. Analysis of DNA content revealed a high recovery of diploid hybrids, although several triploids and a single tetraploid hybrid were also obtained, the latter being stable under laboratory conditions, although it reduced its ploidy to “2n” after mouse passage [79].

The first hybrids generated between different *Leishmania* spp. were obtained in experimental crossings between *L. infantum* LLM-320 (expressing HYG resistance gene) and *L. major* Friedlin V1 (expressing SAT resistance gene) strains in permissive *Lu. longipalpis* sandflies. Genetic analysis of multiple allelic markers indicated that progeny was a complete genomic hybrid, although in these experiments, the rate of hybrid formation was lower than that observed in the intraspecies *L. major* crossing experiments. The resulting hybrids had inherited one allele from each parental line according to mendelian inheritance, i.e., the parasites had the multiple copies in tandem of the visceral species and the single copy of the cutaneous species. The ploidy diversity of the hybrid progeny was variable (2n, 3n, and 4n) and stable. However, the infective behavior to develop skin and/or visceral lesions depended on the dominance of genes from the parental strains. Thus, the ploidy dominance of heterozygous hybrids from *L. infantum* prevented the development of skin lesions, while the ploidy dominance of genes from *L. major* reduced the visceralization of the hybrid strain. Finally, one question raised, but not solved in this work, was the fertility of interspecific hybrids [80].

The first microscopic visualization of fluorescent hybrids of *L. donovani* was performed by Sadlova et al. (2011). In this study, hybridization of two different *L. donovani* strains, LV9 and LEM3804, expressing the antibiotic resistance genes hygromycin and neomycin, together with genes coding the fluorescent proteins GFP and RFP, respectively, was carried out. Recovery of the yellow fluorescent hybrids resulting from mating was localized in the semi-digested blood content of the midgut of both permissive and non-permissive flies two days after feeding. Microscopic identification of the hybrid clones was confirmed by cytometric studies, but the authors were not able to isolate hybrids for further genetic analysis [81].

**Figure 2:**
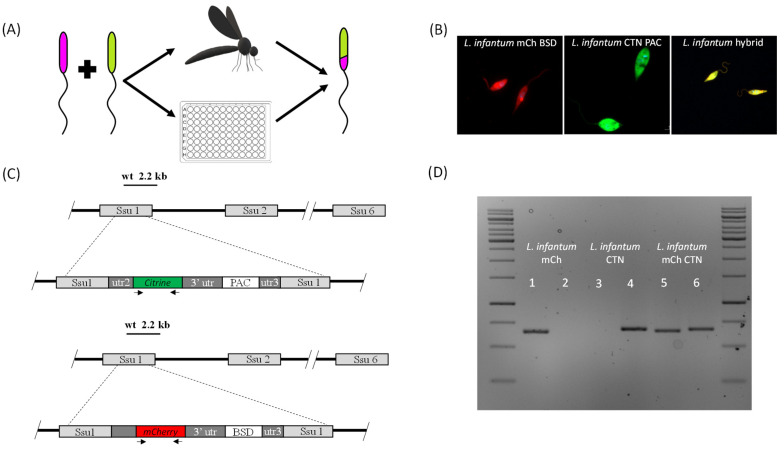
(**A**) Schematic representation of hybrid generation using parental mixtures in the vector insect or *in vitro* using microplates; (**B**) example of two parents used to obtain an intraclonal hybrid between *L. infantum* BCN150 mCherry BSD and *L. infantum* BCN150 CTN PAC; (**C**) schematic showing the integration of pLEXSY plasmids, which allow the expression of fluorescent proteins and antibiotic resistance genes, at the 18S RNA locus; (**D**) specific PCR amplification of the gene encoding fluorescent proteins in the parental parasites (lines 1, 2, 3, and 4) and in the hybrid (lines 5 and 6) [82].

In 2014 Calvo-Álvarez and co-workers isolated for the first time intraclonal *L. infantum* hybrids, which confirmed the ability of these parasites to reproduce by self-fertilization. For this purpose, the authors prepared two stably-transfected parental strains; *L. infantum* CHR+, which constitutively expressed mCherry and was resistant to blasticidine (BLA), and *L. infantum* CTN+, which expressed citrine and was resistant to hygromycin (HYG). Hybrids were recovered from *Ph. perniciosus* flies, and displayed yellow phenotype with double antibiotic resistance (Figure 2). The Mendelian inheritance of the four parental markers was confirmed by PCR. DNA content showed that the ploidy of the hybrid progeny was “3n” in contrast to the “2n” in both parental clones. These hybrids were used in infectivity experiments in BALB/c mice, showing reduced virulence throughout the progression of the disease. Parasites rescued from infected spleens had yellow phenotype, grew in the presence of the two selection antibiotics, and conserved the original “3n” DNA content [82].

More recently, whole genome sequencing was used to analyze the hybrid progeny resulting from experimental intraspecific crosses between *L. major* dLV39c5 × *L. major* Friedlin FV1 [79], from interspecific crosses between *L. infantum* LLM-320 × *L. major* Friedlin FV1 [80], and from ad hoc crosses made for the first time between two strains of *L. tropica*: *L. tropica*/Kub/SAT × *L. tropica* L747/HYG obtained from different geographical origins. Double drug-resistant hybrids recovered from the latter cross between *L. tropica* strains, formally demonstrated the mating competence of members of this species. Moreover, the genetic sequences of the hybrid progeny of *L. major* or *L. tropica* showed a high number and a wide genomic distribution of the heterozygous alleles, which provided a clear evidence of recombination events in *Leishmania*. Moreover, the existence of backcrossing hybrids demonstrated the presence of recombination events, allowed calculating recombination frequencies, and made possible, for the first time, the construction of physical maps of recombination breakpoints in these species [36,83]. The recombination frequencies of *L. major* and *L. tropica* backcrosses were similar to those calculated for *T. brucei* and averaged 1 crossover per 1.5 Mb [84]. However, the mating compatibilities of the experimental hybrids resulting from intra-specific crosses were reduced compared to the parental strain, while the hybrid progeny obtained from *L. major* × *L. infantum* interspecies crossing appeared to be infertile [83].

A recent result demonstrates that hybrid formation in the laboratory can be performed *in vitro* in the absence of the vector. Sacks’ group successfully obtained hybrids from two transgenic strains of axenic promastigotes of *L. tropica* (*L. tropica* L747 RFP-Hyg and *L. tropica* MA37-GFP-Neo). The frequency of hybrid formation *in vitro* was substantially lower than that found in flies, and the genomic stability was maintained after several passages in both fly and culture. SNP marker analysis and whole genome sequencing showed that the clones obtained were complete hybrids. The ploidy of some of the hybrids obtained was close to “2n”, although most were polyploid, either “3n” or “4n” [85]. In a recent paper on *in vitro* hybridization between species of the *L. mexicana* complex, several crosses have been attempted unsuccessfully to study genetic exchange through extracellular vesicles, in axenic cocultures of promastigotes, in infections with bone marrow-derived macrophages, as well as *in vivo*. However, by using fluorescent strains of *L. amazonensis*, intraclonal hybrids within the parasitophorous vacuoles of macrophages have been obtained. Interestingly, these vacuoles can harbor several amastigotes of these species, unlike what it is observed with Old World species, where each vacuole harbors one amastigote, thus preventing physical contact between parasites [86].

## 4. Genetic Inheritance of kDNA

How and from whom extranuclear kinetoplast DNA (kDNA) is inherited in hybrid progeny after mating are other important questions that need to be answered. The kDNA is a unique intricate disc-shaped mesh of extranuclear DNA exclusively found in kinetoplastids, which consists of thousands of intertwined DNA rings structured into conserved maxicircles, and a heterogeneous population of minicircles [87]. Minicircles are the most abundant component (5000–10,000 molecules per cell) and encode guide RNAs, which are involved in editing nascent mitochondrial RNAs [88]. In contrast, maxicircles have a much larger perimeter, but are much less abundant than minicircles (several dozen per cell), and are equivalent to the mitochondrial DNA of other eukaryotes [87]. Maxicircles contain two ribosomal RNA genes, fourteen protein-coding genes homologous to mitochondrial genes typical of other eukaryotes, four genes of unknown function, and some RNA guides [89]. Replication of kDNA occurs synchronously with nuclear DNA during cell division. Components of the kDNA must be decatenated from the network before DNA replication and after this, it is catenated back into the nascent mesh [90] before segregation.

Although kDNA apparently behaves as a compact mass of DNA, the inheritance of maxicircles and minicircles in the progeny that results from mating events is controversial. Early polymorphism analyses of DNA maxicircles in *T. brucei* hybrids, suggested that the inheritance of hybrid progeny was uniparental, as it occurs with mitochondrial DNA in other eukaryotes [91,92,93]. However, hybrids with biparental maxicircles were later described in *T. brucei*, especially in the early growth stages of hybrid progeny resulting from a genetic cross [47,94,95]. One possible explanation for this surprising fact is that, although the inheritance of maxicircles is biparental, after several mitotic divisions, one of the parental genotypes dominates and becomes fixed in subsequent generations at the expense of the other [94]. This is probably explained by the genetic drift that occurs when the number of DNA molecules is small and a minimum number of generations have elapsed after crossing [96]. However, unlike maxicircles, when the composition of minicircles in *T. brucei* was examined by restriction and hybridization analyses, the minicircle networks of the hybrid progeny appeared to be heteroplasmic, thus concluding that the inheritance of minicircle kDNA was biparental [95,97]. The biparental inheritance of mitochondrial DNA from maxicircles and minicircles after mating suggests that not only the fusion of gamete cytoplasm is necessary, but also the fusion of each of the parental mitochondria [47] (Figure 1B).

Studies of kDNA gene exchange in other trypanosomatids are less numerous than in *T. brucei*, and less conclusive too. Several reports have shown that the inheritance of the maxicircle was uniparental in both *T. cruzi* and *Leishmania*, although the recovery of hybrids during the earliest stage of their generation has not been yet explored [64,78,79,80,98,99]. On the other hand, the inheritance of the minicircles is biparental, although evidence is still scarce in both species [99].

## 5. Conclusions

Kinetoplastids belong to the eukaryote supergroup considered an early diverging branch of the eukaryotic tree. Despite their ancestral origin, these eukaryotes contain the complete toolkit for meiosis. True sex, incorporating meiosis with generation of haploid gametes or gamete-like cells has been definitely described in *T. brucei*, but not in other species of trypanosomatids. These processes are difficult to detect because sex does not appear to be an obligatory stage of their life cycle, there is no obvious sexual dimorphism and sometimes the mating competent forms are confined to one stage of the life cycle.

A second unanswered question is why many of the hybrids of the progeny of *Leishmania* are aneuploid after fusion of nuclei. *Leishmania*, despite being considered “mainly diploid”, is constitutively aneuploid in mosaic for many of its chromosomes. In addition, no segregation of its complete chromosome set can occur, and viable polyploid 3n and 4n hybrids can be produced. Mosaic aneuploidy results mainly from asymmetric chromosome allocation during mitosis, and can be puzzling when genetic exchange between aneuploid progeny takes place. The fusion of these two “parental” aneuploid cells gives rise to aneuploid cells, which will lead to reduction and redistribution of chromosomes in daughter cells, thereby resulting in “chromosome shuffling”. The question is whether the combination of mosaic aneuploids is a selective advantage for the parasite and how often sexual or parasexual processes occur.

The formation of new and more virulent clades that can differentiate into new species containing characteristics of the two parents has been described between the non-pathogenic strain of *T. b brucei* and the virulent *T. b. gambiense* type 2 or *T. b. rodhesiense*. Another example is represented by the hybrids formed after mating visceralizing forms of *L. donovani* or *L. infantum* with the cutaneous strain of *L. major*, or by the examples of hybrid DTUs in *T. cruzi*. This points to the importance of this process in the development of new species.

## Data Availability

Data sharing not applicable.
